# Novel ultra-stretchable and self-healing crosslinked poly (ethylene oxide)-cationic guar gum hydrogel

**DOI:** 10.1186/s13036-023-00376-2

**Published:** 2023-10-16

**Authors:** Sergio Alberto Bernal-Chávez, Sergio Alcalá-Alcalá, Zainab M. Almarhoon, Aknur Turgumbayeva, Eda Sönmez Gürer, Ma. De Los Dolores Campos-Echeverria, Hernán Cortés, Alejandra Romero-Montero, María Luisa Del Prado-Audelo, Javad Sharifi-Rad, Gerardo Leyva-Gómez

**Affiliations:** 1 Departamento de Ciencias Químico-Biológicas, Universidad de Las Américas Puebla, Ex-Hda. de Sta. Catarina Mártir, 72820 Puebla, Cholula Mexico; 2 Laboratorio de Tecnología Farmacéutica, Facultad de Farmacia, Universidad Autónoma del Estado de Morelos, Cuernavaca, Morelos Mexico; 3 Department of Chemistry, College of Science, King Saud University, P. O. Box 2455, 11451 Riyadh, Saudi Arabia; 4 Higher School of Medicine, Al-Farabi Kazakh National University, Almaty, Kazakhstan; 5 School of Pharmacy, JSC “S.D. Asfendiyarov Kazakh National Medical University”, Almaty, Kazakhstan; 6 Department of Pharmacognosy, Faculty of Pharmacy, Sivas Cumhuriyet University, Sivas, Turkey; 7 Departamento de Farmacia, Facultad de Química, Universidad Nacional Autónoma de México, 04510 Ciudad de Mexico, Mexico; 8 Laboratorio de Medicina Genómica, Departamento de Genómica, Instituto Nacional de Rehabilitación Luis Guillermo Ibarra Ibarra, 14389 Ciudad de México, Mexico; 9 Tecnologico de Monterrey, Campus Ciudad de México, 14380 Ciudad de Mexico, Mexico; 10 Facultad de Medicina, Universidad del Azuay, Cuenca, Ecuador

**Keywords:** Cationic guar gum, Hydrogel, Poly(ethylene oxide), Soft tissues, Tissue engineering, Wounds

## Abstract

Hydrogels are three-dimensional structures with specific features that render them useful for biomedical applications, such as tissue engineering scaffolds, drug delivery systems, and wound dressings. In recent years, there has been a significant increase in the search for improved mechanical properties of hydrogels derived from natural products to extend their applications in various fields, and there are different methods to obtain strengthened hydrogels. Cationic guar gum has physicochemical properties that allow it to interact with other polymers and generate hydrogels. This study aimed to develop an ultra-stretchable and self-healing hydrogel, evaluating the influence of adding PolyOX [poly(ethylene oxide)] on the mechanical properties and the interaction with cationic guar gum for potential tissue engineering applications. We found that variations in PolyOX concentrations and pH changes influenced the mechanical properties of cationic guar gum hydrogels. After optimization experiments, we obtained a novel hydrogel, which was semi-crystalline, highly stretchable, and with an extensibility area of approximately 400 cm^2^, representing a 33-fold increase compared to the hydrogel before being extended. Moreover, the hydrogel presented a recovery of 96.8% after the self-healing process and a viscosity of 153,347 ± 4,662 cP. Therefore, this novel hydrogel exhibited optimal mechanical and chemical properties and could be suitable for a broad range of applications in different fields, such as tissue engineering, drug delivery, or food storage.

## Introduction

Hydrogels (HGs) are three-dimensional structures with specific characteristics that make them widely used in biomedical applications, such as tissue engineering scaffolds, drug release platforms, and wound dressings [6, 8, 20, 32]. HGs can be prepared from different materials; however, natural materials are widespread for preparing HGs since they fit the requirements for "green" and sustainable development [12]. Moreover, natural polymers have played an essential role in producing biomaterials due to their biocompatibility, nontoxicity, biodegradability, ease of availability, and low cost [5, 17]. Polysaccharides, such as cationic guar gum (CGG), are abundant, biocompatible, biodegradable, and bioactive [4, 12, 21]. CGG is the modification of naturally occurring guar gum (GG) in which hydroxyl groups are substituted by hydroxypropyltrimonium groups [16]. The net positive charge causes CGG to be readily crosslinked (CL) with anions, such as Sodium tetraborate decahydrate (borax). CGG has been used as an excellent non-gelling thickener and enhancer of viscosity and volume [16]. Furthermore, CGG has remarkable antibacterial properties, and compared to regular GG, CGG has superior solubility and thermal stability [16]. However, HGs constituted only by CGG have poor mechanical properties [10], limiting their application in various fields. On the other hand, poly(ethylene oxide) (PolyOX) is a water-soluble resin based on a long-chain nonionic polyethylene oxide polymer with low toxicity and poor absorption. It imparts lubricity to end products, resulting in a silky sensation. Moreover, PolyOX can be used to fabricate HGs that are highly water retentive, can be extruded, injection molded, or casted. Likewise, PolyOX may create films that are flexible and resistant to oil and greases [14], properties that can be used to create HGs for several sectors such as biomedicine, food, cosmetic, and pharmaceutical, among others. PolyOX is a homopolymer that can form hydrogen bonds between the ether oxygen in its structure and hydroxyl and carboxyl groups of other polymers, such as polysaccharides [7, 36]. Consequently, the preparation of CL CGG-based HGs by adding PolyOX is an effective solution for improving the performance of individual polysaccharides.

This study hypothesizes that a CL CGG HG could modify its mechanical and rheological properties by including PolyOX to obtain a more stretchable biomaterial capable of spreading over large areas of wounds. In this study, we compared the mechanical and chemical behavior of CGG-PolyOX with CL CGG HG and a simple mixture (non-CL) of CGG and PolyOX (CGG + PolyOX). The obtained HG presented semi-crystalline, ultra-stretchable, and self-healing properties compared to CL CGG and the non-CL mixture of CGG and PolyOX HGs. Furthermore, to our knowledge, there is no prior information about the elaboration of CGG-PolyOX HGs and the study of modifications of the mechanical properties triggered by the PolyOX addition.

## Materials and methods

### Materials

CGG (N-Hance™ CG13, guar hydroxypropyltrimonium chloride, medium charge density, medium nitrogen content, and high molecular weight) was donated by Ashland®. PolyOX with a molecular weight 200,000 (PolyOX™ WSR N80) was obtained from DuPont™ (Delaware, USA). Borax was obtained from Chemical Products Mardupol (Morelos, Mexico). The solutions were prepared using water from a Milli-Q® (MQ) filtration system (Millipore, Billerica, MA, USA).

### Fabrication of the cationic guar gum-poly(ethylene oxide) hydrogel

HGs at different ratios of CGG and PolyOX were generated. Table 1 describes the quantities and concentrations used for diverse mixtures. The next procedure was followed for all the HGs. First, we added an amount of CGG powder into 50% deionized water, needed to prepare 100 g of HG at 21 °C, and stirred at 100 rpm for 30 min. Then, the pH was adjusted with a 15% citric acid solution (180 μL) to promote the dissolution of the CGG polymer, and the stirring was maintained for 10 min more.

**Table 1:** The concentration of polymers and the amount of crosslinker used to fabricate hydrogels

Mixtures	CGG (% w/v)	PolyOX (%w/v)	Borax (mg)
M1	1.0	0.5	50.0
M2	1.0	1.0	50.0
M3	1.0	2.0	50.0
M4	---	1.0	50.0
M5	1.0	---	50.0
M6	1.0	1.00	---

On the other hand, we dissolved PolyOX powder in the rest of the deionized water at the same temperature and speed. Once both polymers were mixed, we added the PolyOX solution to the CGG solution and mixed it for 15 min at 500 rpm. Next, for mixtures (M1-M5), a volume equivalent to 50 mg of borax of a 1% solution was added and mixed for 5 min at 500 rpm. Finally, the HG was left to rest for 24 h at 25 $^\circ{\rm C}$. For M4 and M5, the procedures to prepare the CGG and PolyOX solutions were omitted, considering that these mixtures only contained one of the polymers based on Table 1. However, we added borax in the same way as the mixtures M1-M3.

#### Rheological behavior, spreadability, and pH degradability of HGs

We analyzed the rheological behavior of all HGs using the following procedure. First, we evaluated the viscosity of the different HGs through a combination of visual assessment and viscosity measurements taken at regular intervals. Additionally, we determined the free spreadability factor of the HGs by taking a mass of HG weighing 5.0 ± 0.5 g, placed on a smooth surface, and allowed to spread freely for 10 min. Subsequently, three diameter measurements were taken at various positions using a digital caliper. We calculated the spreadability factor (Sf, mm^2^/g) by using the equation ${\mathrm{S}}_{\mathrm{f}}={}^{\mathrm{A}}\!\left/ \!{}_{\mathrm{W}}\right.={}^{\left({}^{{\mathrm{d}}^{2}\uppi }\!\left/ \!{}_{4}\right.\right)}\!\left/ \!{}_{\mathrm{W}}\right.$, where: *d* = average diameter; *W* = total weight (g), and *A* = total area (mm^2^). Moreover, 3.0 g of each HG (except M4 and M6) were placed in different buffer solutions (pH 4.0, 6.8, and 9.0) at 25 $^\circ{\rm C} ,$ and the weight after one h in contact with the solution was measured. We determined the HG's degradability percentage by considering the initial and final weights.

### Characterization of the optimized hydrogel

Once we found the HG containing PolyOX with the best rheological and mechanical properties to be extended without breaking easily, adequate degradability percentage, and spreadability factor, it was characterized by the following tests by using lyophilized HGs (Sects. "Differential scanning calorimetry (DSC)" – "Infrared spectroscopy" and "Swelling test") and non-lyophilized HGs (Sects. "pH influence on rheological performance", "Rheological behavior" and "Self-healing, extensivity, and visual performance"). The CL HG (CGG-PolyOX) was compared with CL CGG and with an HG prepared as CGG-PolyOX but without crosslinking (CGG + PolyOX).

#### Differential scanning calorimetry (DSC)

We tested HG samples of 10 mg using a differential scanning calorimeter (DSC, TA Instrument™, Q20) to determine the presence of thermal events associated with amorphous and crystalline materials and contrast with those obtained in the X-ray diffraction (XRD) studies. The samples were positioned in aluminum hermetic crucibles. We performed measurements under a 50.0 ml/min nitrogen flow in the temperature range from 125 $^\circ{\rm C}$ to 300 $^\circ{\rm C}$ at a heating rate of 10 $^\circ{\rm C}$/min. Before heating, the samples were kept in equilibrium with an isotherm at 20 $^\circ{\rm C}$ for 2 min.

#### Scanning electron microscopy (SEM)

We determined the morphology and microstructure of HG samples in a scanning electron microscopy (Evo 10, Zeiss, Germany) operating at 5 kV. Samples of HGs were fixed using carbon tape on pin stubs of aluminum and sputter-coated with a thin layer of gold.

#### X-ray diffraction (XRD)

To confirm a change in the crystallinity of CGG-PolyOX, we used an XRD-Bruker® D8-Advance (Ettlingen, Germany) X-ray diffractometer to analyze the XRD pattern of raw materials, CGG-PolyOX, and CL CGG at room temperature. We performed the sample analysis with the Cu-Kα radiation at the wavelength of 1.54 Å working at 40 kV in the scanning angle range of 2θ = 5 $^\circ$−40 $^\circ$ with a Lynxeye silicon strip detector.

#### Infrared spectroscopy

To evaluate the possible chemical changes associated with the crosslinking and the interactions between polymers, the infrared spectra were acquired by a Cary 630 FTIR equipment (Agilent Technologies®, Santa Clara, CA, USA). Samples of raw materials, CGG-PolyOX, and CL CGG, were placed on a horizontal diamond cell of attenuated total reflection (ATR) to record the infrared spectrum. Measurements represented an average of 150 scans with a resolution of 16.

#### pH influence on rheological performance

In order to analyze the stretching behavior of HGs at different pH values, we performed a macroscopic test to determine the time it took for a 290 mg object to be displaced by 30 mm within samples of the HGs at pH 3.0, 7.5, and 9.0, as described by [31]. We conducted this evaluation at a temperature of 25 $^\circ{\rm C}$ and in triplicate to ensure accuracy. Subsequently, the displacement factor (D_f_) was calculated using the equation: ${\mathrm{D}}_{\mathrm{f}}={}^{\mathrm{W}}\!\left/ \!{}_{(\mathrm{d}*\mathrm{t})}\right.$, where: *d* = distance (mm); *W* = object weight (mg), and *t* = average time (min).

#### Swelling test

Crosslinking of polymer chains can modify the response to the HGs; therefore, we measured the swelling profile. For the swelling test, 0.2 g of sample was put into a meshed diffusor and was introduced into the water at 25 $^\circ{\rm C}$ with a constant stirring at 50 rpm and taking different samples in the interval of 0.01–8 h. After each sample, we determined the weight of the previously blotted with a filter paper, and finally, the sample was returned to the water. This procedure was repeated as many times as necessary until the increase in swelling capacity became constant. The test was carried out in triplicate. Finally, both profiles (CGG-PolyOX and CL CGG) were analyzed based on Peleg, first-order, and pseudo-second-order models for swelling kinetics [18, 22] according to the following models:

- Peleg: t/[Q_t_ – Q_e_] = k_1_ + k_2_t
- First order: ln (Q_e_ − Q_t_) = ln(Q_e_) − k_1_t
- Pseudo-second order: t/ Q_t_ = [ 1/k_2_Q_e_] + t/Q_e_

Where Q_e_, Q_t_, k_1_, and k_2_ are defined as swelling content at equilibrium, swelling content at the time (t), and swelling kinetic constants, respectively.

#### Rheological behavior

The rheological and viscosity measurements of CGG-PolyOX, CL CGG, CGG + PolyOX, and healed CGG-PolyOX were carried out in a DV2T Viscometer (Brookfield AMETEK®, USA) by using the link hanging spindle SC4-34 with an SRC (Shear Rate Constant) of 0.28 N used to convert the set rpm to shear rate and calculate shear stress and with a viscosity range of 30–600,000 cP. The measurements were done by comparing time and viscosity, shear rate and shear stress, and shear rate and viscosity. All the analyses were performed at 25$^\circ{\rm C}$, 2.5 rpm, and triplicate. All the flow curves obtained were analyzed with different rheological models: Power law (η = m.γ ^−n^), Bingham (τ = τ_0_ + η.γ), and Casson (τ^0.5^ = τ_0_^0.5^ + η^0.5^. γ ^0.5^). The terms from the equations are as follows: *τ* is the shear stress (Pa), *γ* is the shear rate (s^−1^), *η* is the viscosity (Pa. s), *τ*_*0*_ is the yield stress (Pa), *m* is associated with the viscosity obtained for the shear rate of 1 s^−1^, and *n* is the flow index (dimensionless) indicating the non-Newtonian or Newtonian behavior [1, 15].

#### Self-healing, extensivity, and visual performance

The extensibility and flexibility of the HGs were assessed using a modified semiquantitative macroscopic method based on the protocol described by [23]. We securely clamped a 20.0 ± 2.5 g sample of CGG-PolyOX between a pair of jaws and allowed it to undergo elongation under the influence of gravity at a height of 93 cm. We meticulously recorded the precise elongation moment before any HG rupture. The experiments were conducted with and without prior sample stretching to compare the outcomes.

A 10 g portion of CGG-PolyOX was initially placed on a plastic dish to evaluate the self-healing properties. A deep cut, measuring 40 ± 1.0 mm in diameter, was then created in a diametrical manner using a glass rod with a width of 5.0 ± 0.5 mm and a depth of 0.5 ± 0.1 mm. Subsequently, we determined the time required for the cut line to close without any imperfections on the surface completely. The technique provided an assessment of the self-healing capability of the HG. After that, the healed HG disk was held up to check its healing ability.

#### Statistics

The Minitab® XVII computer program was used to perform statistical analysis. The influence of different factors on the behavior of CGG-PolyOX was performed by analysis of variance (ANOVA), while the difference between means was assessed using the t-student test and confidence intervals, with a significance level set at *p* ≤ *0.05.*

## Results and discussion

### Rheological behavior, spreadability, and pH degradability of HGs

**Fig. 1:**
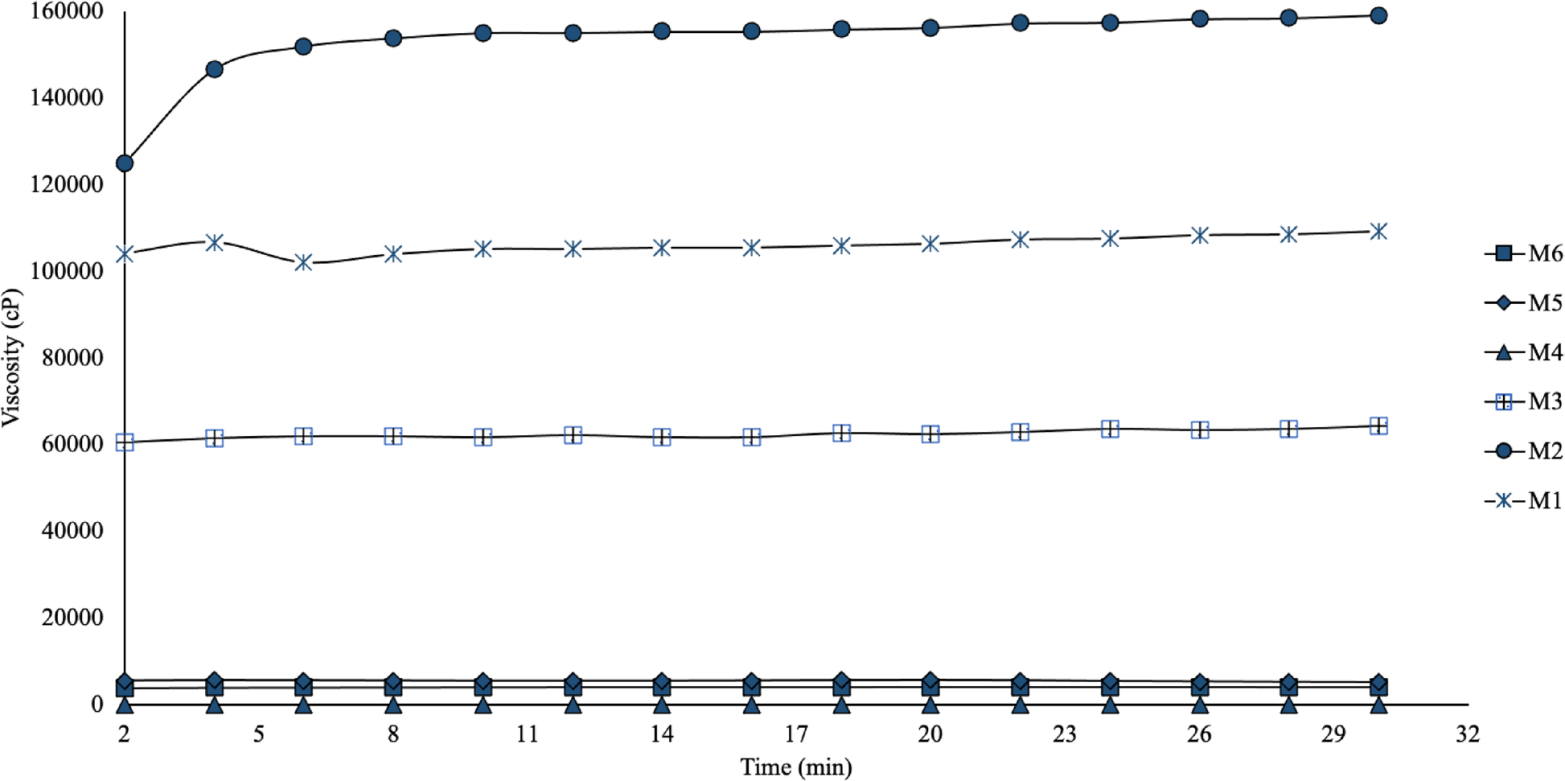
Viscosity kinetics of the different HGs. The viscosity for all HGs remains constant over time. M2 and M4 exhibited the highest and lowest viscosity, respectively. The composition of the HGs is described in Table 1

We evaluated three concentrations of PolyOX to find its optimal concentration and assess the effect of crosslinker on the rheological and flexibility properties. Table 2 shows the viscosity, spreadability, density, and percentage of degradation results at different pH for the different HGs prepared. As seen in Fig. 1, the HG designated M2, with a concentration of 1.0% PolyOX, 1.0% CGG, and crosslinker, exhibited the highest viscosity concerning the rest of the HGs. The difference is significant compared to M6, whose concentrations of both polymers are identical, but without a crosslinker (*p* < 0.05). The crosslinker increased the crosslinking of the CGG chains, allowing for a more mechanically resistant HG. In addition to the hydrogen bonds produced between the hydroxyl groups of the CGG and the polar groups in the PolyOX and the intermolecular forces (such as Van der Waals forces, ion–dipole, and dispersion) between both polymers, the difference between M2 and M6 could be attributed to the consumption of hydroxyl groups of galactomannan backbone in the formation of covalent bonds with borax [27]. The presence of crosslinker in M4 did not significantly affect (*p* > 0.05) the viscosity of the HG when compared to the obtained for the HG that only contained PolyOX at 1.0% and whose value was 17.64 ± 1.03 cP. Then, the crosslinking effect in PolyOX chains, observed in HGs containing CGG, is practically null.

**Table 2:** Properties of the different HGs of CGG and PolyOX associated with the change in concentration of PolyOX and the presence of crosslinker

	**M1**	**M2**	**M3**	**M4**	**M5**	**M6**
CGG (% w/v)	1.0	1.0	1.0	---	1.0	1.0
PolyOX (%w/v)	0.5	1.0	2.0	1.0	---	1.0
Borax (mg)	50.0	50.0	50.0	50.0	50.0	---
Viscosity (cP)	105,947 ± 1,074	153,347 ± 4,662	62,384 ± 563	18.45 ± 0.09	5,380 ± 80	4,004 ± 41
Sf (mm^2^/g)	379.72 ± 6.52	323.48 ± 3.15	459.22 ± 12.52	589.86 ± 55.60	472.72 ± 29.80	571.67 ± 35.40
Density (g/ml)	0.972 ± 0.223	0.961 ± 0.041	0.992 ± 0.129	1.002 ± 0.023	0.966 ± 0.225	1.034 ± 0.452
Degradability at pH = 9.0 (%)	25.84 ± 4.55	36.25 ± 4.02	46.74 ± 6.12	---	33.06 ± 11.91	---
Degradability at pH = 6.8 (%)	55.21 ± 9.53	66.65 ± 3.57	76.39 ± 13.43	---	34.52 ± 14.28	---
Degradability at pH = 4.0 (%)	64.09 ± 8.89	72.13 ± 3.99	85.50 ± 8.68	---	59.05 ± 11.66	---
Physical appearance

On the other hand, in the HGs that contain constant concentrations of crosslinker and CGG (M1-M3), the viscosity rises when changing from 0.5 to 1.0% of PolyOX (Table 2, M1 and M2). However, the viscosity decreases again when PolyOX is added at 2% (M3). This effect is because a higher concentration of PolyOX exerts a more significant plasticizing effect on the HG, making it more fragile and, thus, more fluid. This behavior can be corroborated by the physical appearance of each HG in an inverted cell (Table 2, M1-M3). Finally, when comparing the viscosity and appearance of M2 and M5 (whose only difference is the presence of PolyOX), it can be supposed that including PolyOX (M2) allows obtaining an HG with an improved viscosity. This property could impact the HG application, giving it better manageability and flexibility.

In the case of Sf, the value of M2 was lower than the other HGs, which is related to the increase in its viscosity. The other HGs showed the opposite behavior of what was observed in viscosity. For example, M2 had a lower Sf than M1. But for M3, the Sf was higher,which means that the increase in the concentration of PolyOX makes it spread more easily, associated with the plasticizing effect of the polymer. M4 exhibited the highest Sf of all the HGs due to its lower viscosity, which makes it spread easily when allowed to spread freely.

Regarding the behavior of the HGs at different pH, an increase in pH causes a lower percentage of HG degradation in the analyzed cases. Decreased degradation percentage is associated with the HG acquiring greater rigidity at a basic pH, which allows for keeping the polymeric chains united for a longer time. Likewise, the basic pH decreases the solubility of the CGG related to its cationic properties, which increases the hydrophobicity of HG. Furthermore, an alkaline pH will allow HG to maintain its mechanical properties and lower degradation percentage.

In the case of the HGs M1-M3, the increase in the PolyOX concentration augmented the degradation percentage for the three buffer solutions. This behavior is because a higher concentration of PolyOX implies more significant plasticity of the HG [3], which causes a greater degree of diffusivity to the medium. Likewise, the hydrophilicity of PolyOX due to the high content of hydroxyl groups causes a greater formation of hydrogen bonds with water, allowing greater HG solubilization in the medium. HGs M4 and M6 were discarded because their rheological properties were not functionally adequate for their intended use, and specifically, M4 dissolved rapidly in all buffer solutions tested.

### Characterization of the optimized hydrogel

Once we analyzed the properties of the different HGs shown in Table 2, M2 was chosen as the best due to its physical appearance, viscosity, and adequate flexibility. We compared the HG M2 (CGG-PolyOX) with two control HGs, CL CGG and a non-CL mixture of CGG and PolyOX (CGG + PolyOX). The concentrations of polymers in control HGs are the same as in CGG-PolyOX.

#### Differential Scanning Calorimetry (DSC)

**Fig. 2:**
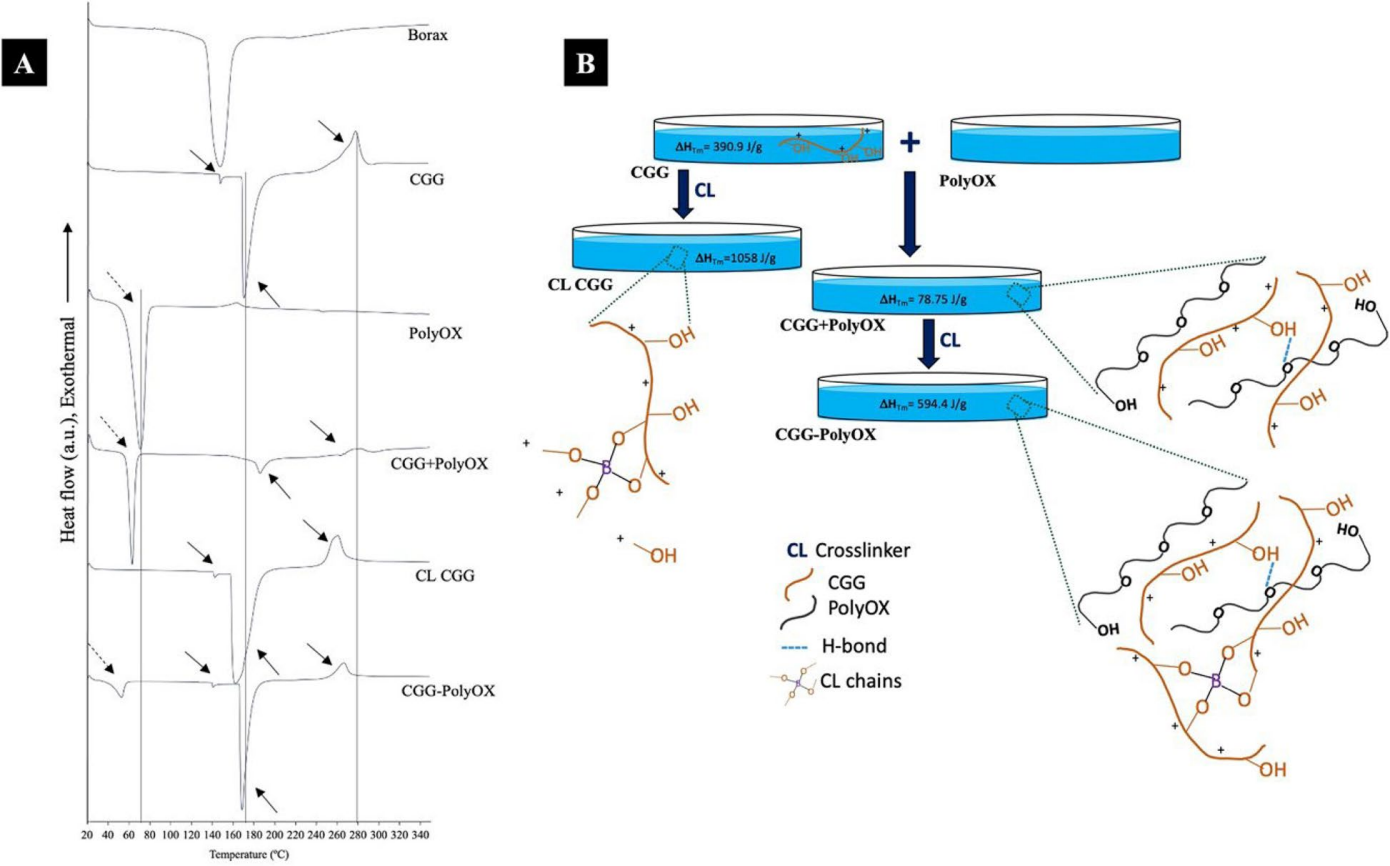
**A** Thermal behavior of the optimized CGG-PolyOX based on DSC. Solid arrows are thermal events related to CGG (peaks 2, 3, and 4); dashed arrows are thermal events associated with PolyOX (peak 1). **B** Diagram of CGG-PolyOX hydrogel formation and interactions with the crosslinker. The ΔH is associated with the thermal event (Tm) of CGG (peak 3)

Figure 2A exposes the corresponding thermograms for CGG-PolyOX, CL CGG, CGG + PolyOX, and the raw materials. DSC analyses demonstrated that the melting point (Tm) of PolyOX was approximately 68.47 $^\circ{\rm C}$ (Table 3) and that it agrees with those reported for various polyethylene oxides [33]. The Tm (Peak 1) for CGG-PolyOX was 51.27 $^\circ{\rm C}$ (dashed arrows), representing a decrease of approximately 17 $^\circ{\rm C}$, while for CGG + PolyOX, this decrease was approximately 7 $^\circ{\rm C}$. In this respect, the presence of the crosslinker had a more significant impact on the Tm of the HGs than PolyOX. The area under the melting endotherm curve is related to the crystallinity in the specimen [26], and as mentioned in the XRD analysis, the incorporation of the crosslinker generated HGs with a reduced crystallinity. It can be observed that the CGG-PolyOX Tm associated with PolyOX decreases in intensity and transforms into a broader Tm, which supports our results in XRD.

**Table 3:** Thermal properties of the HGs. The table exposes the results associated with peaks from left to right detected in the thermograms. The red markers in the sparklines only represent cells with values for each column; for example, the three markers in the sparkline for peak 1 do not include CGG and CL CGG

	**Peak 1**		**Peak 2**		**Peak 3**		**Peak 4**	
	**Tm**	**ΔH**	**Tg**	**ΔH**	**Tm**	**ΔH**	**Td**	**ΔH**
	$^\circ{\rm C}$	**J/g**	$^\circ{\rm C}$	**J/g**	$^\circ{\rm C}$	**J/g**	$^\circ{\rm C}$	**J/g**
**PolyOX**	68.47	305.7	---	---	---	---	---	---
**CGG**	---	---	147.51	4.428	170.60	390.9	277.25	203.8
**CL CGG**	---	---	140.92	3.519	159.31	1058	259.51	195.1
**CGG + PolyOX**	61.55	609.7	---	---	185.36	78.75	273.44	78.66
**CGG-PolyOX**	51.3	88.29	139.09	2.496	165.31	594.4	264.95	101.6
**Sparkline**								

On the other hand, the CGG presented three peaks (solid arrows): two endothermic peaks at 147.51 $^\circ{\rm C}$ associated with a glass transition temperature (Tg) and at 170.60 $^\circ{\rm C}$ corresponding to a Tm, and an exothermic peak at 277.25 $^\circ{\rm C}$ (Td) which can be associated with a vigorous thermal decomposition (with the release of gaseous products having a higher enthalpy of formation). Other GG derivatives also show this type of exothermic event of decomposition where there is not any endothermic onset and, as a result, exotherm associated with this enthalpy of formation masks the observation of endotherm related to the energy required for decomposition [28].

As can be seen in the sparkline of peak 2, the presence of a crosslinker decreases Tg by 4.5% for CL CGG; in the case of CGG-PolyOX, the reduction in Tg was 5.7%. The CGG + PolyOX thermogram did not show a Tg. Thus, it can be inferred that the addition of PolyOX in the absence of the crosslinker generates an HG with higher crystallinity, considering that the Tg is usually a characteristic presented by amorphous or semi-crystalline materials [19]. On the other hand, for peak 3, the Tm decreases for CL CGG, increases in CGG + PolyOX, and decreases again in CGG-PolyOX but above CL CGG; this same behavior was presented for Td in peak 4. This finding suggests that the crosslinker alone has a more significant effect on the thermal changes in HGs than when it is combined with PolyOX. For these two peaks, the ΔH is lower for CGG + PolyOX than CGG-PolyOX, which implies that the CL system requires more energy to generate the associated thermal events, namely, the possible breaking of the bonds between the borax and the CGG chains and the hydrogen bonds between both polymers. However, this phenomenon is the opposite for peak 1, where ΔH is higher in CGG + PolyOX (609.7 J/g) than in CGG-PolyOX (88.29 J/g). Since this peak is a thermal event assigned to the PolyOX and peaks 3 and 4 to CGG, it can be established that the crosslinking caused by borax only occurs mainly at the level of the hydroxyl groups from CGG and not with those present in PolyOX (Fig. 2B).

#### Scanning electron microscopy (SEM)

**Fig. 3:**
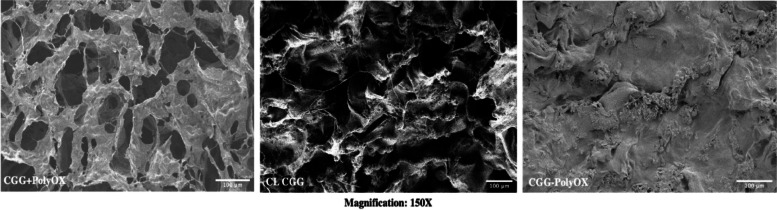
Morphology of the optimized CGG-PolyOX, CL CGG, and CGG + PolyOX based on SEM

Micrographs of the CGG-PolyOX, CGG + PolyOX, and CL CGG are presented in Fig. 3. It can be observed that CGG-PolyOX exhibits a smooth surface; meanwhile, CL CGG possesses a coarser surface. After adding the crosslinker, the surface of CGG + PolyOX changes from a rough network with an irregular appearance and a high degree of porosity to a more compact and smoother appearance with slightly uneven regions. This result can be explained by the formation of a chemically crosslinking network in the HG skeleton.

Comparing the morphology of CL CGG and CGG-PolyOX with CGG + PolyOX, it can be concluded that crosslinking promotes the formation of an HG with more compact surfaces and, additionally, the presence of PolyOX in CGG-PolyOX generates a more compact HG structure. It could be due to factors such as a higher polymeric concentration compared to CL CGG, the linear polymeric structure of PolyOX that can promote the formation of more ordered matrices, and, finally, the chemical affinity promoted by the interaction by hydrogen bonds between the CGG and the PolyOX.

### X-ray diffraction (XRD)

**Fig. 4:**
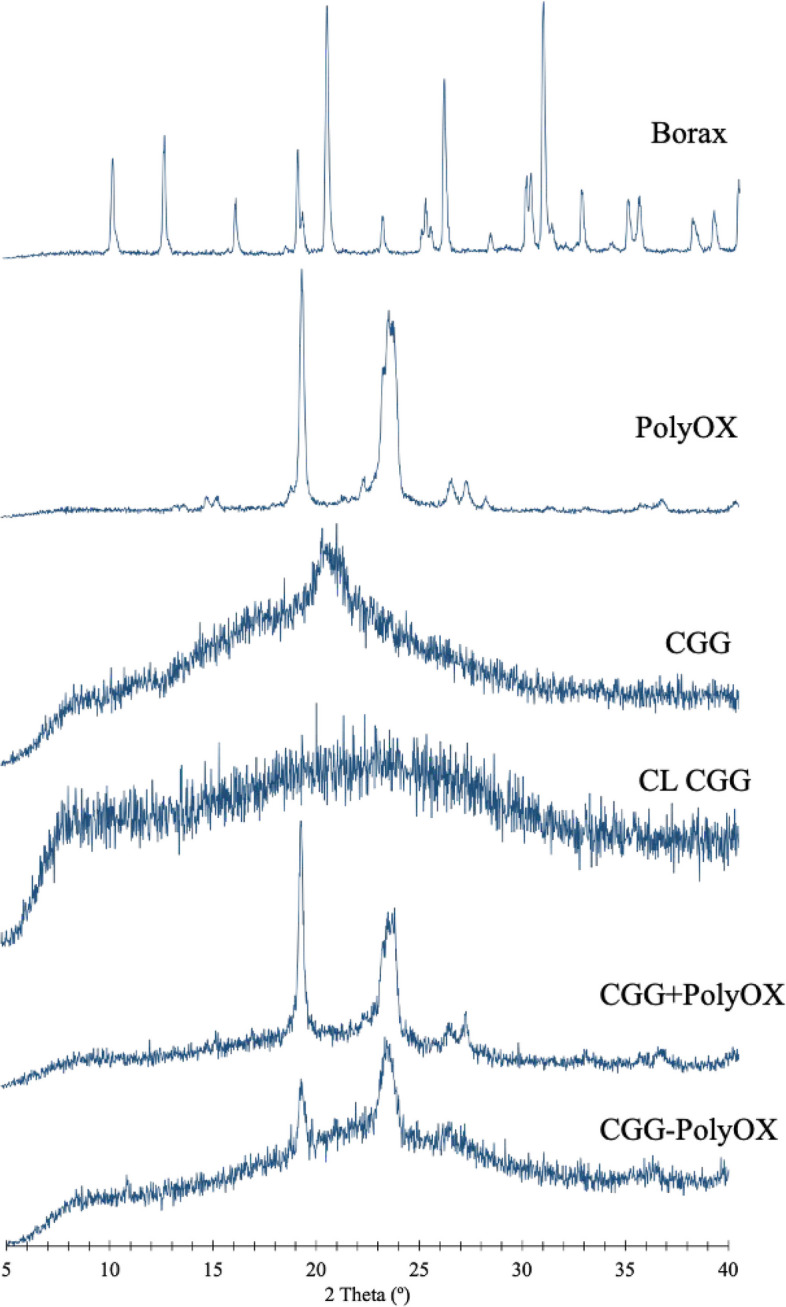
Diffraction patterns of the optimized CGG-PolyOX

From the curve of CGG in Fig. 4, the broad peak known as the "bun-like peak" is caused by the amorphous phase in CGG. However, the sharpest peak (2θ = 20.46 $^\circ$) on the "bun-like peak" indicates that CGG exhibits a very small crystallinity. Such characteristics coincide with previously published results, where the CGG showed a characteristic peak at 2θ = 20.38 $^\circ$, which decreased after fluoridation (C. [30]. In this case, a remarkable reduction in crystallinity was observed for CL CGG after the addition of the crosslinker. On the other hand, PolyOX presents a crystalline structure represented by two high-intensity diffraction peaks at 19.23 $^\circ$ and 23.34 $^\circ$ and weak reflections at 13.61 $^\circ$ and 27.32 $^\circ$ [2]. The signal is also diminished in the CGG-PolyOX system, where the bun-like peak disappears, and the two peaks associated with PolyOX become irregular and slightly broader. In this regard, it can be inferred that incorporating borax in the polymeric matrix leads to the disorganization of the chains, which generates an HG with semi-crystalline properties.

#### Infrared spectroscopy

**Fig. 5:**
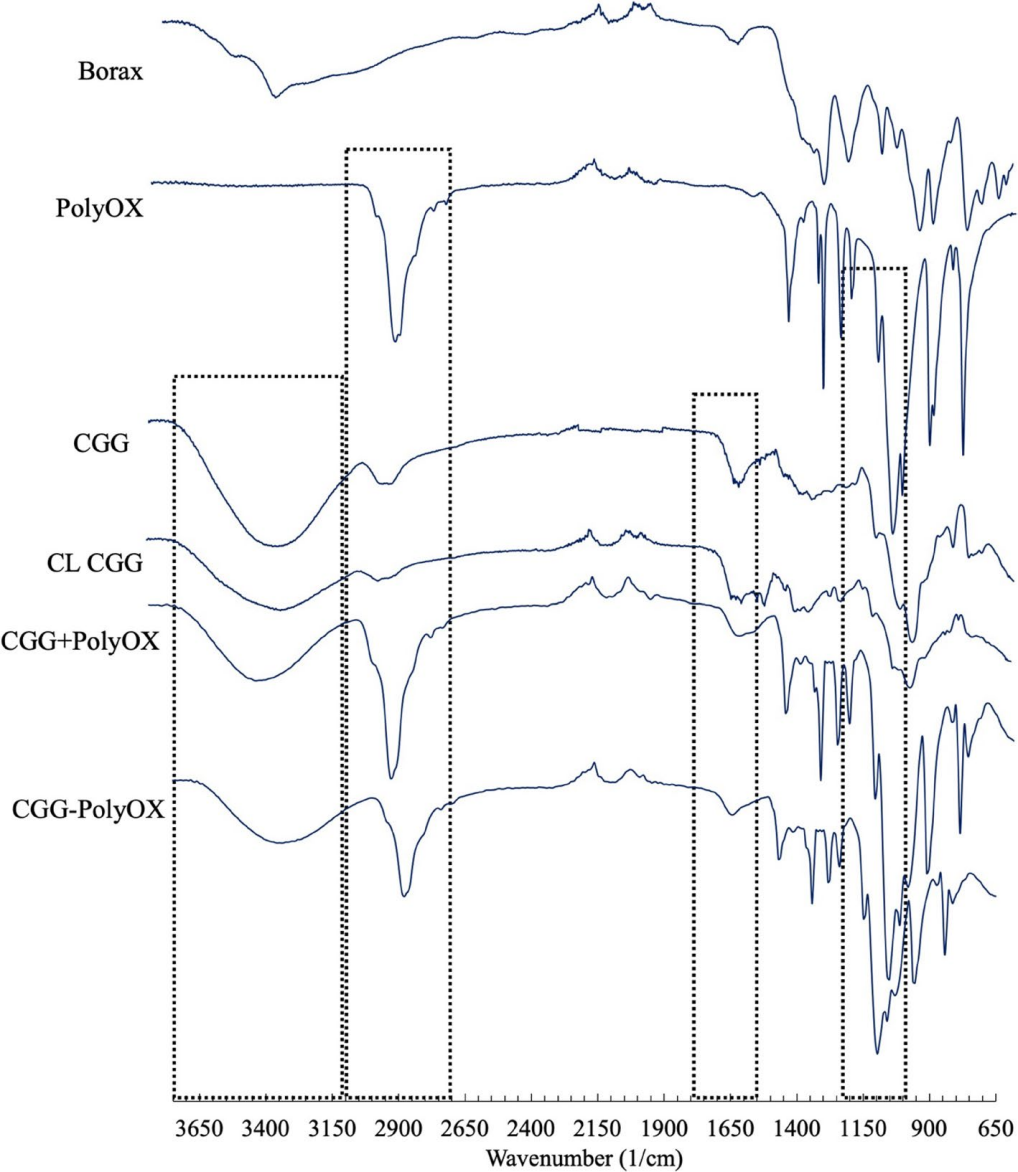
FTIR spectra. The dashed boxes represent the four main regions analyzed for the functional group vibrations in raw materials and in HGs. From left to right: O–H, C-H, the cationic polymer region of CGG, and C-O and C–O–C stretching vibrations

As part of evaluating the chemical interaction between the various materials within the structure of the HGs, we analyzed the infrared spectra of the three HGs, and the raw materials are presented in Fig. 5. Infrared spectra of CGG-PolyOX, CL CGG, and CGG + PolyOX revealed characteristic vibrational signals associated with similar vibration modes. The FT-IR bands between ν = 3700 and 3000 cm^−1^ and the shoulder-shaped band centered at ν = 2900 cm^−1^, depicted within dashed boxes, assigned to the O–H stretching mode of the hydrogen-bonded hydroxyl and C-H (aliphatic) groups in both polymers, respectively [16]. The C-H signal in CGG becomes broader when the presence of borax crosslinks it. In the case of CGG + PolyOX, the peak shows characteristics more like PolyOX. However, this signal also decreases intensity and shifts towards lower wavenumber values in CGG-PolyOX. The primary evidence for the cationic polymer absorption can be observed in the 1700–1400 cm^−1^ region. Instead, in CL CGG, there is a band around ν = 1700 cm − 1 (C = O stretching) indicative of formed ester for the effect of the chemical interaction between the crosslinker and the cationic region of CGG. Conversely, systems containing PolyOX (CGG + PolyOX and CGG-PolyOX) present a smoothed signal in the same region, which may be associated with the interaction in the HGs by hydrogen bonds between the cationic portion of CGG and PolyOX. The absorption bands at 1146, 1056, and 1010 cm^−1^ are associated with C-O and C–O–C stretching vibrations. These signals in CGG + PolyOX and CGG-PolyOX show greater similarity with the PolyOX signals. However, these are broader and can be attributed to hydrogen bond interactions in both polymers. The broad peak at 961 cm^−1^ is assigned to the C–C-N coupled stretching in the trimethyl ammonium group [16].

#### Influence of pH

**Fig. 6:**
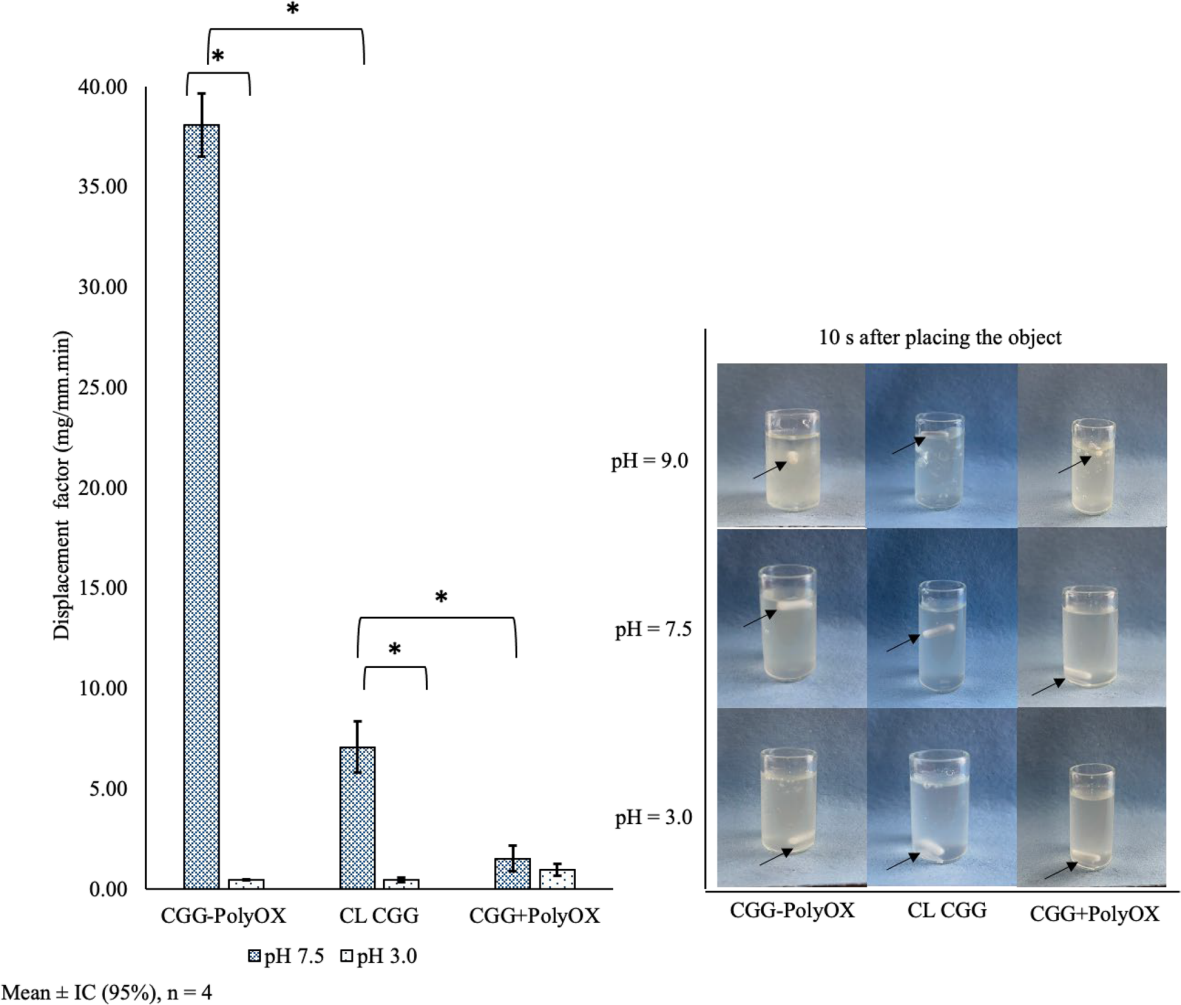
The behavior of CGG-PolyOX, CL CGG, and CGG + PolyOX at different pH. The graph on the left shows the displacement factor (Df) of the HGs at pHs 7.5 and 3.0, and on the right side, the physical behavior of an object (black arrow) when it is dropped freely inside of each HG at pH 3.0, 7.5 and 9.0. The photo represents the position of the object 10 s after placing it

As discussed earlier, pH is crucial in influencing the prepared HGs' behavior, particularly regarding their rheological properties. We assessed the impact of pH on HGs, examining the variations associated with different pH values. In Fig. 6, the left side graph illustrates the observed displacement factor (D_f_) of an object freely dropped onto HGs with pH 7.5 and 3.0. The D_f_ values are higher for all three HGs at pH 7.5, with CGG-PolyOX exhibiting a significantly higher D_f_ (*p* < 0.05) than the other two HGs. The right diagram of Fig. 6 visually demonstrates this phenomenon, where 10 s after placing the white object, it sinks to the bottom of the container in the case of CGG + PolyOX and even remains on the surface in the case of CGG-PolyOX. On the other hand, at pH 3, the object is found at the bottom after 10 s due to the disruption of the polymeric structure of the HGs caused by the acidic nature of the medium, rendering the systems more fluid. For pH 9.0, the opposite behavior is observed, as the object remains on the surface in all cases. Consequently, the measurement of Df for this pH was not performed. This is attributed to the fact that an alkaline pH induces a more rigid structure in the CGG-containing HGs, resulting from increased viscosity, hydrophobicity, and electrostatic repulsion of the CGG cationic chains. The stability of the ester bond explains the behavior of the CL-CGG sample. The rate of hydrolysis in esters is directly related to pH, limiting their usefulness to a pH range of 5–10 [29]. This explains the behavior of greater mechanical resistance at pH 9.0 and decreased performance with decreasing pH.

#### Swelling test

HGs are polymeric materials that can swell in water and retain a significant fraction of water within their structure without dissolving [35]. The swelling behavior of a polymer depends on several factors, such as the hydrophilic–hydrophobic interactions and the degree of crosslinking of the network [13].

**Fig. 7:**
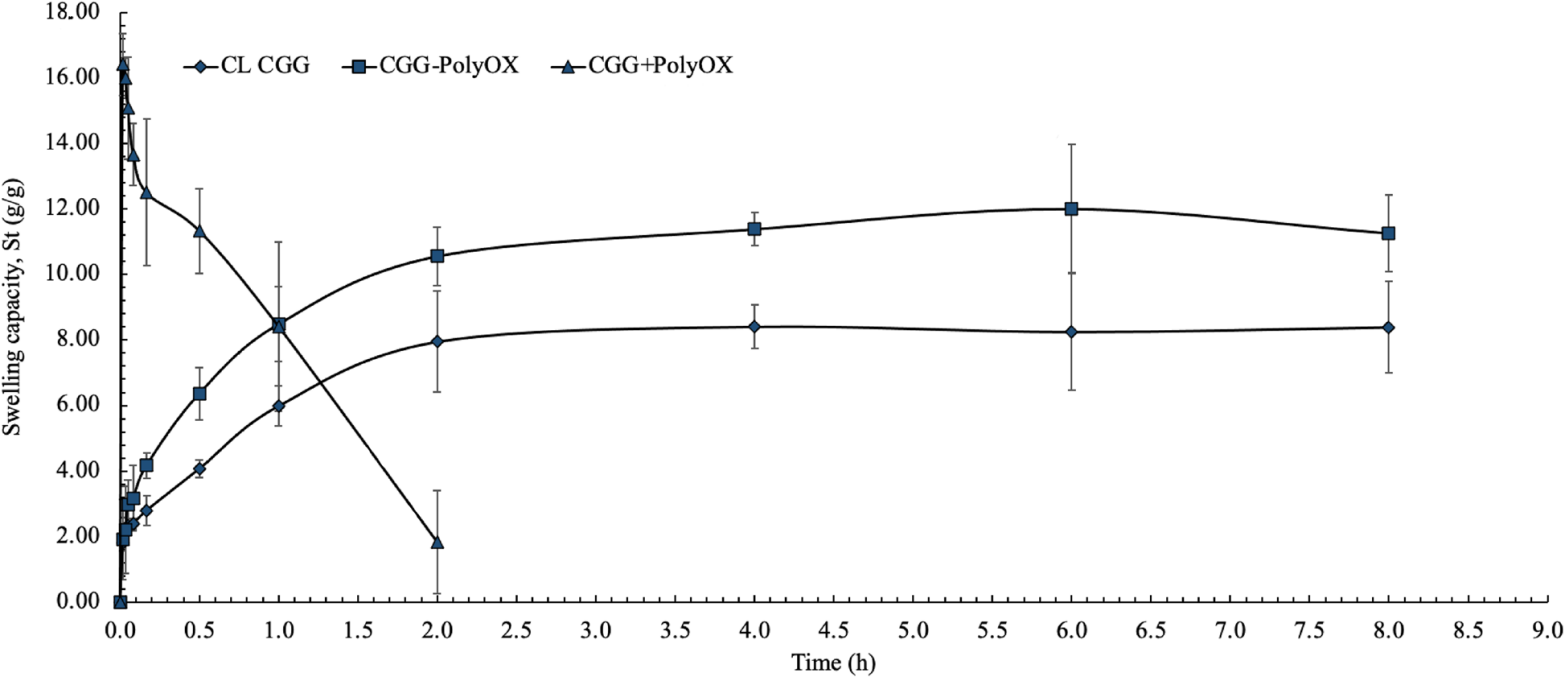
Swelling kinetic profile of the optimized CGG-PolyOX (mean ± CI, 95%, *n* = 3)

The CGG-PolyOX and CL CGG presented a significantly different swelling profile from the first minutes of analysis concerning the CGG + PolyOX, as exposed in Fig. 7. Based on the Voigt model, the Se and r are the maximum water-holding capacity and the time required to reach 0.63 of the equilibrium swelling (K. [35]. The Se of CGG-PolyOX and CL CGG were 10.56 g/g and 7.95 g/g, achieved after two h, with r of 31.3 min and 31.9 min, respectively, while the CGG + PolyOX showed a maximum water-holding of 16.42 g/g in just 1 min. Based on the models for swelling kinetics [18, 22], the pseudo-second-order kinetic fits most accurately to the swelling process for CGG-PolyOX and CL CGG with determination coefficients (R^2^) of 0.9969 for both HGs (Table 4) and Q_e_ of 11.8906 and 8.6580, respectively. These values are similar to those found in the Voigt model. As described, k_2_ in the Peleg model and k_1_ in the first-order model are constants corresponding to the slope of the profile. In both cases, these constants present negative values for CGG + PolyOX, which justifies a process of dissolution of the HG in the test fluid and not a swelling, unlike CGG-PolyOX and CL CGG, where their slopes are positive.

**Table 4:** Fitting data of the HGs with different swelling models

Model	Parameters	CGG-PolyOX	CL CGG	CGG + PolyOX
Peleg	R^2^	0.8685	0.9060	0.9674
	k_1_	-0.5648	-0.9574	-0.0372
	k_2_	1.2113	2.6237	-0.0520
First order	R^2^	0.9749	0.9470	0.1439
	k_1_	1.4696	1.1676	-2.8972
Pseudo-second order	R^2^	0.9969	0.9969	0.9056
	k_2_	4.2908	4.0526	-1.9755
	Q_e_	11.8906	8.6580	-0.9679

Of the two CL HGs, CL CGG presented a significantly lower swelling from the first 10 min compared to CGG-PolyOX. This difference is associated with the presence of PolyOX, which is a high-water retentive polymer.

Although both HGs, CGG + PolyOX and CGG-PolyOX, present the same concentration of PolyOX, water uptake during the first minutes is significantly higher in CGG + PolyOX. In this case, crosslinking plays an essential role in the swelling of both HGs, converting the CGG-PolyOX polymeric matrix into a less hydrophilic material. Additionally, it can be assumed that the presence of hydrophobic regions in the CGG and PolyOX backbones causes a higher intermolecular interaction due to weak forces such as dispersion and dipole-induced dipole. Those attractive intermolecular forces are maximum in regions with higher crystallinity within the HG. In addition, CGG + PolyOX has a more porous morphology, which makes it easier to take up water compared to the more compact morphology of CGG-PolyOX.

#### Rheological behavior

**Fig. 8:**
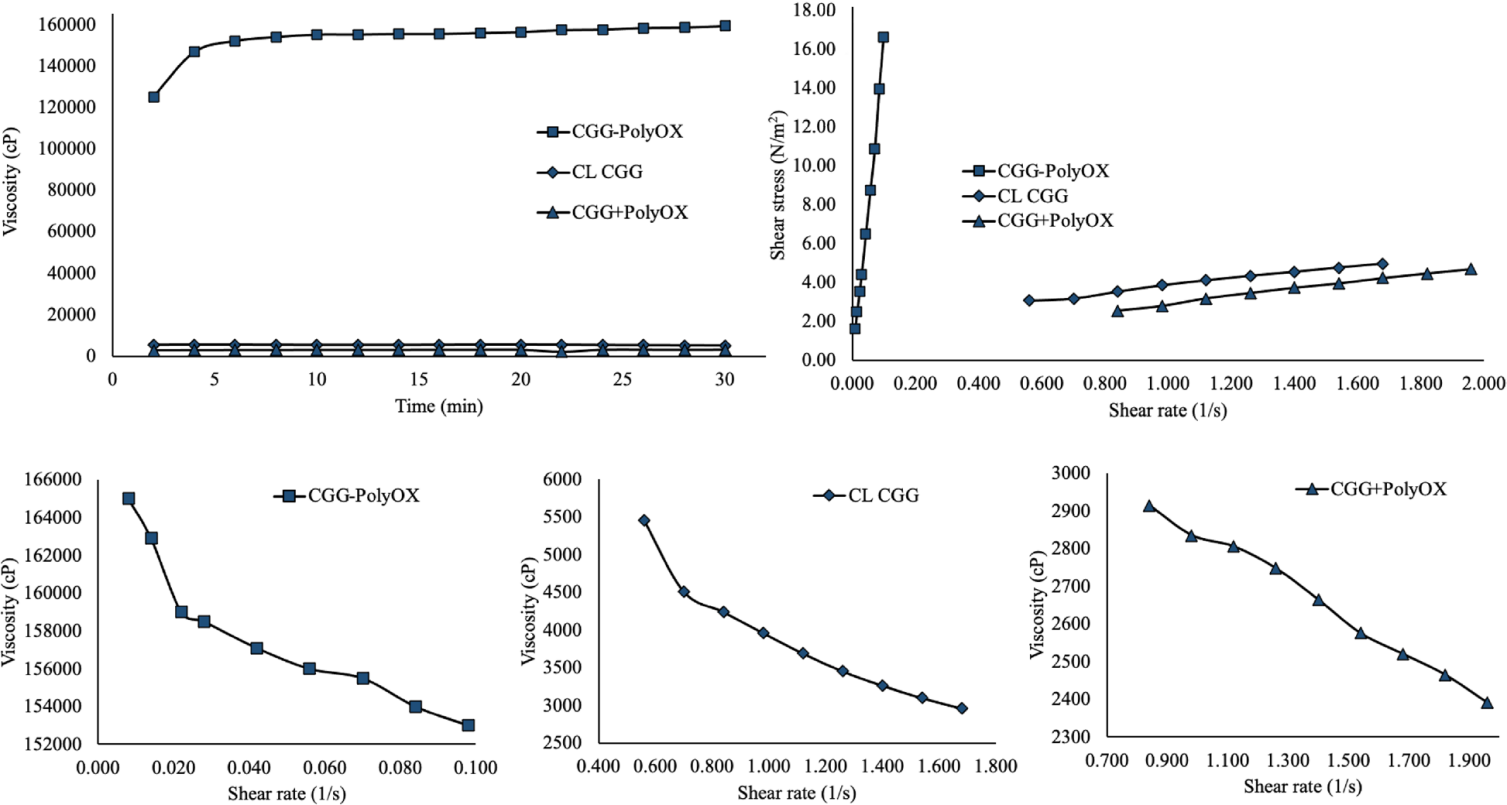
Rheological behavior of CL CGG, CGG + PolyOX, and CGG-PolyOX. The upper plots show the change in viscosity concerning time (left) and the change in shear stress concerning shear rate (right), while the lower plots represent the change in viscosity concerning shear rate

Figure 8 shows the rheological profiles of CL CGG, CGG + PolyOX, and CGG-PolyOX. As previously mentioned, the viscosity values for all HGs remained constant in the studied range, thus classifying them as time-independent systems. CGG-PolyOX presented significantly higher values of viscosity (*p* < 0.05) concerning CL CGG and CGG + PolyOX (upper left plot). The HGs exhibited a reduction in viscosity with an increase in shear rate (lower plots). Additionally, the upper right graph combination confirmed that all three HGs demonstrated non-Newtonian fluid properties, specifically displaying pseudoplastic behavior. The data in Table 5 show that the HGs exhibit pseudoplastic properties, with the flow index "n" being less than 1. The "n" moves from values around one, typical of Newtonian behavior, to values between 0 and 1 for a non-Newtonian pseudoplastic system [9]. The "n" values are correlated with the degree of pseudo-plasticity, with smaller values leading to a marked degree of shear thinning [15]. It has been reported that HGs from GG and CL CGG with a boron derivative behave as typical non-Newtonian shear-thinning fluids, showing "n" values of 0.2786 and 0.4935, respectively [25]. This behavior and values are similar to that observed for CL CGG (n = 0.5295). Concerning this, the influence of PolyOX on the rheological properties of the polymer matrix can be evidenced by a lower "n" value for CGG-PolyOX followed by CGG + PolyOX.

**Table 5:** Fitting data of the HGs with different rheological models

Model	Parameters	CGG-PolyOX	CL CGG	CGG + PolyOX
Power law: η = m.γ ^−n^	R^2^	0.9800	0.9909	0.9647
	m	14,3317	3,893	2,844
	n	0.0289	0.5295	0.2331
Bingham: τ = τ_0_ + η.γ	R^2^	0.9948	0.9912	0.9954
	η	0.0060	0.5596	0.5164
	τ_o_	0.0007	-1.1437	-0.4965
Casson: τ^0.5^ = τ_0_^0.5^ + η^0.5^. γ ^0.5^	R^2^	0.9948	0.9927	0.9967
	η	0.0064	1.1686	0.7012
	τ_o_	1.6 × 10^–5^	1.2627	0.1800

The pseudoplastic behavior is a desirable property for the semisolid dosage forms. It enables the HG to flow easily at high shear rates, facilitating topical administration. On the other hand, at low shear rates, the material reverts to a higher consistency, thus recovering its original rheological properties. As presented in Table 3, the values recorded for all models' "R2" parameter ranged between 0.9647 and 0.9967, indicating that those rheological models fit adequately with the experimental data obtained. However, the Casson model works slightly better for all three HGs. The "m" parameter is higher for CGG-PolyOX, which indicates that the polymeric matrix is stronger and has a higher resistance to shear-induced destruction.

#### Extensivity and self-healing

Self-healing HGs are a promising strategy in biomedical applications such as tissue engineering, wound healing, and drug delivery, controlling their responses by external stimuli like pH, temperature, and pressure. As mentioned, PolyOX can increase the flexibility of other polymers that lack it [14], which allows the creation of materials with potential applications in biomedicine.

**Fig. 9:**
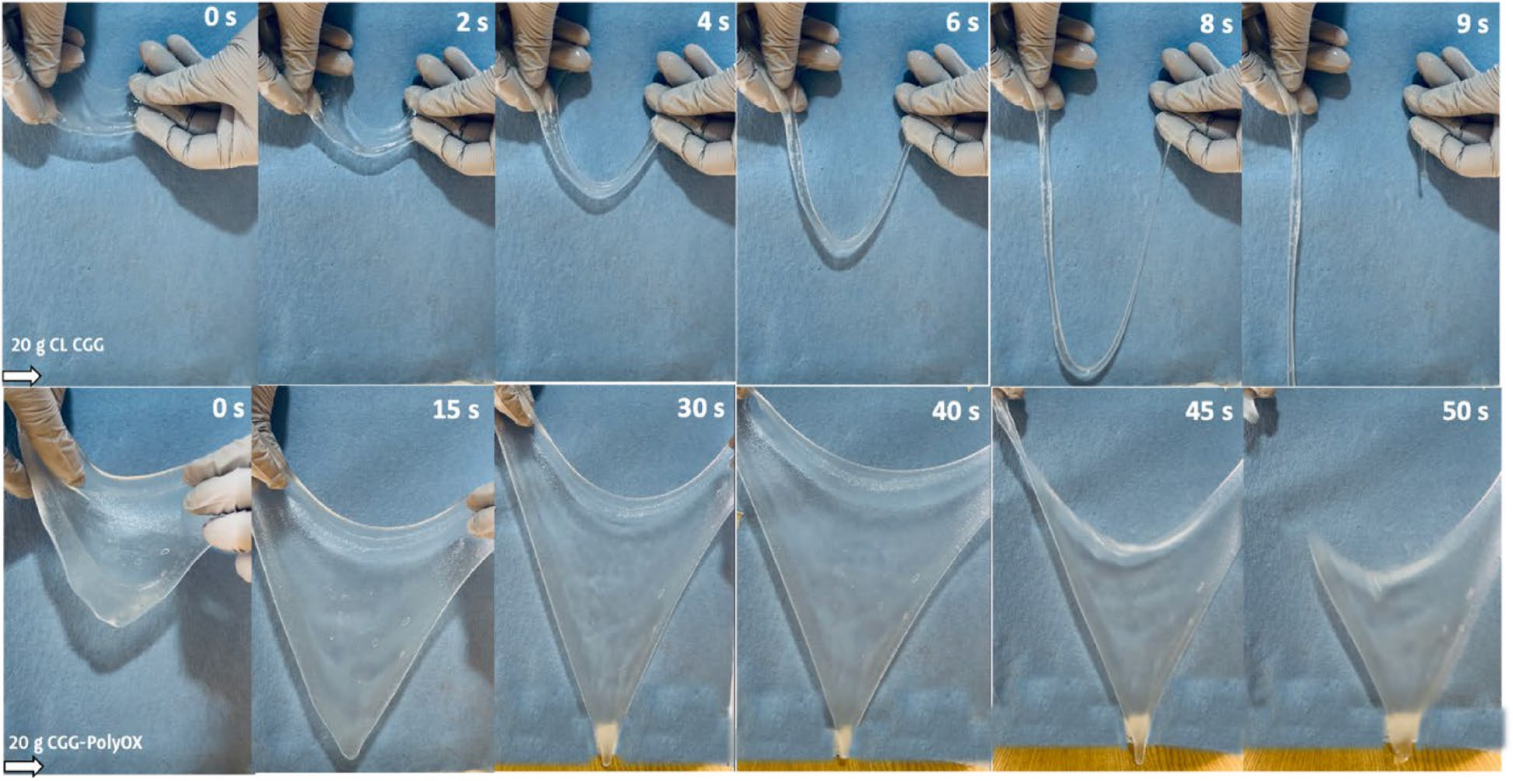
Semiquantitative flexibility of CL CGG (upper) and CGG-PolyOX (lower). The sample taken for the study was 20 g, and the time indicated in the upper right represents the time elapsed from the extension of the HG to its rupture due to gravity

In this case, 1.0% PolyOX was the adequate concentration to obtain an HG with the ideal mechanical properties, evaluated in a semiquantitative test. The aforementioned semi-crystalline properties of CGG-PolyOX support this situation. Specifically, it has been observed an augmentation in the extent of crystalline regions to enhance the rigidity and resilience of polymer-based materials. Conversely, a decrease in the size of these regions, accompanied by a greater abundance of amorphous regions, leads to an increase in flexibility. Furthermore, this heightened flexibility is associated with weak intermolecular forces, which facilitate the extension of polymer chains [11, 34]. To corroborate this effect, we performed a macroscopic analysis with a mass of 20 g of CL CGG and CGG-PolyOX to observe the gravitational effect on these materials that elongated freely, and the time to rupture was measured (Fig. 9).

CGG-PolyOX exhibited superior flexibility than CL CGG. While CL CGG resisted for approximately 9 s before breaking, CGG-PolyOX resisted for up to 50 s with an area of about 400 cm^2^ and a mass of 20 g, representing a 33-fold increase in size from its original state. CGG + PolyOX was not analyzed in this test because it is a very fluid HG that cannot be spread between the jaws.

**Fig. 10:**
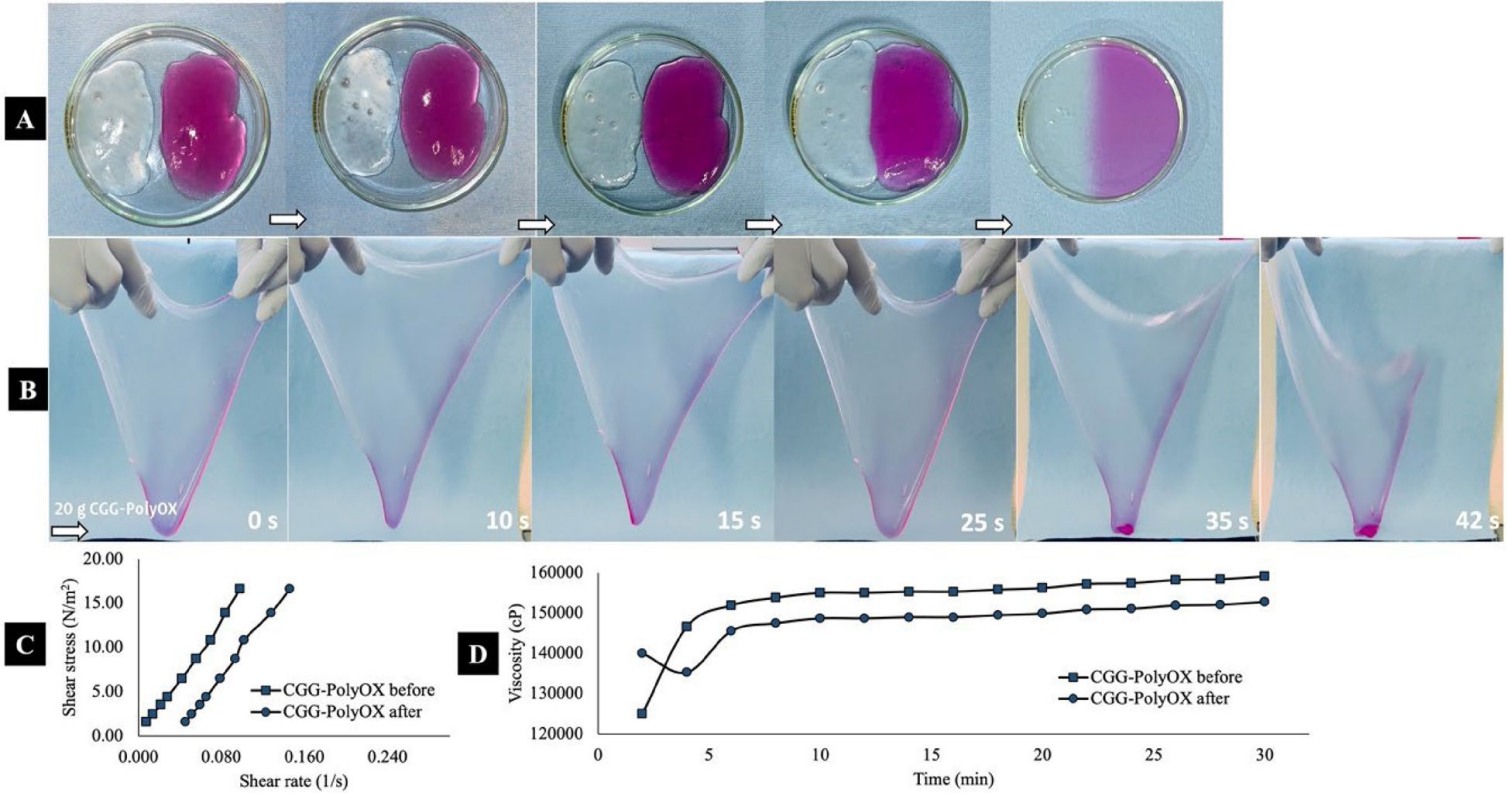
Self-healing behavior **A**, semiquantitative flexibility **B**, and rheological comparison of CGG-PolyOX before and after self-healing **C** and **D**. The sample taken for the study was 20 g, and the time indicated in the lower right represents the time elapsed from the extension of the HG to its rupture due to gravity. CGG-PolyOX has a transparent appearance. However, to evidence self-healing, which occurred in an average of 12.5 ± 2.5 min, the HG was stained with D&C Red No. 33, CI 17200

On the other hand, a self-healing test was carried out to verify if the CGG-PolyOX rupture maintains its rheological and flexibility characteristics after self-healing. As seen in Fig. 10A, CGG-PolyOX can self-heal in an average time of 12.5 ± 2.5 min and remains a material with superior flexibility than CL CGG (Fig. 10B). Based on the conducted analysis, the observed self-healing mechanism of CGG-PolyOx could be primarily attributed to hydrogen bonding interactions between PolyOX chains, CGG chains, and between hydrogen donor and acceptor groups of both polymers. These interactions are enhanced within an aqueous environment, facilitating their potency. Moreover, the substantial presence of ether groups in PolyOX and hydroxyl groups in CGG contributes to the relatively rapid self-healing mechanism (Fig. 2B).

When measuring the time to rupture due to gravity, self-healed CGG-PolyOX revealed a decrease that was not statistically significant (*p* > 0.05) compared to CGG-PolyOX before self-healing. This self-healing behavior of HG is due to the formation of interactions between polymer chains. These interactions can be formed through diverse chemistries and mechanisms, such as dynamic covalent bonds, non-covalent interactions, and multi-mechanism interactions [24]. In this case, the interactions were mainly non-covalent hydrogen bonds. The lower part of Fig. 10 (C and D) exposes the shear stress versus shear rate and viscosity versus time profiles of CGG-PolyOX before and after self-healing. In this case, the self-healed CGG-PolyOX reveals a displacement in both graphs. Although this displacement is not statistically significant (*p* > 0.05) with a 96.8% viscosity recovery after self-healing, it allows inferring that the constant mechanical damage to the HG can cause a change in its rheological properties. Remarkably, the self-healed CGG-PolyOX remains the same pseudoplastic non-Newtonian fluid observed for CGG-PolyOX before self-healing.

As it could observed, the obtained semi-crystalline HG, CGG-PolyOX, presented transparency, a high swelling capacity, remarkable stretchability, and extraordinary extensibility. All these properties demonstrated that the HG has a potential application in biomedical strategies such as controlled delivery and dressings for tissue engineering, among others.

## Conclusions

In this study, we developed and characterized an ultra-stretchable and self-healing HG based on PolyOX and CGG at a 50:50 ratio (% wt/wt), which showed adequate mechanical and chemical properties for a potential application in the biomedical field. The novel HG was obtained by mixing concentrations of 1% for each polymer and 0.05% of borax as a crosslinker, resulting in semi-crystalline, ultra-stretchable, and self-healing properties. The HG exhibited viscosity values of 153,347 ± 4,662 cP, appropriate for wound healing dressings compared to CL CGG and a non-CL mixture of CGG and PolyOX HGs. Likewise, the concentration of PolyOX in the HG influences the ultra-stretchable and self-healing behavior with an extensibility area of approximately 400 cm^2^, which represented a 33-fold increase concerning the HG before being extended and a post-self-heal recovery of 96.8%, respectively. Also, a concentration higher than 1.0% generates more fluid HGs without elastic properties, and lower concentrations produce more viscous but challenging-to-spread HGs; this behavior was equally influenced by the change in pH.

## Data Availability

Yes.
